# Supplementary material for: Real-time dynamics of Plasmodium NDC80 reveals unusual modes of chromosome segregation during parasite proliferation
Source: J Cell Sci. 2020 Jun 30;134(5):jcs245753. doi: 10.1242/jcs.245753 (PMC7340582; doi:10.1242/jcs.245753)
Supplement: Supplementary information [file joces-134-245753-s1.pdf]

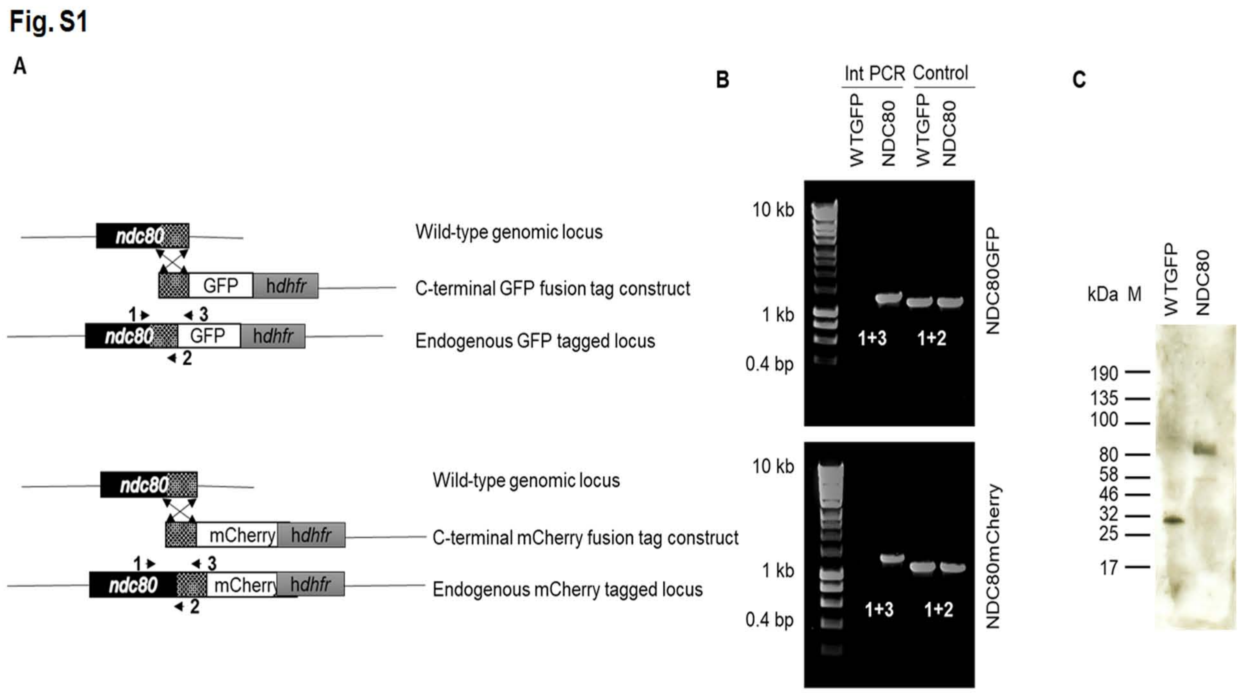

Fig. S1: Generation and genotype analysis of NDC80-GFP/mCherry parasite lines. (A) Schematic representation of the endogenous *Ndc80*, the GFP/mCherry-tagging construct and the recombined *Ndc80* locus following single homologous recombination. Arrows 1, 2 and 3 indicate the position of PCR primers used to confirm successful integration of the construct. **(B)** Diagnostic PCR of NDC80-GFP and WT-GFP parasites using primers IntT259 (NDC80, Arrow 1) and ol492 (for GFP line)/mCherry (for mCherry) (Arrow 3). IntT259 and T2592 (NDC80, Arrow 2) primers were used as control. Integration of the NDC80 tagging construct gives a band of 1269bp (GFP line) and 1335bp (mCherry line) for NDC80 parasite line. For controls, both WT and NDC80 tagged constructs gave a band size of 1153 bp. **(C)** Western blot of NDC80-GFP (96 kDa) and WT-GFP (29kDa) protein to illustrate the presence of intact NDC80-GFP in schizont stage extracts.

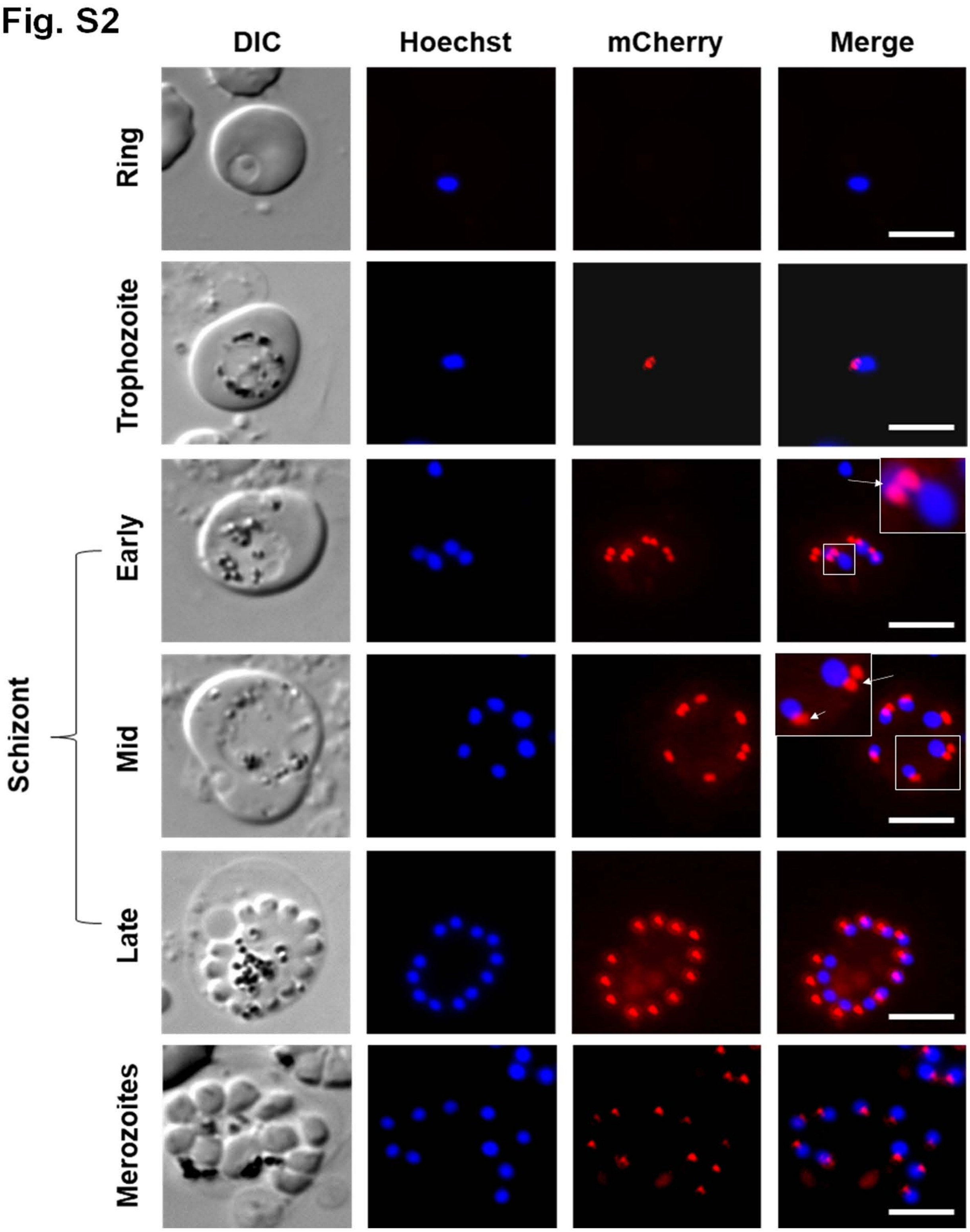

Fig. S2: Expression and location of NDC80-mCherry in asexual blood stages. Fluorescence was detected by live cell imaging. DIC: Differential interference contrast; Hoechst: blue, DNA; mCherry: red, NDC80-mCherry; Merge: Hoechst and mCherry fluorescence. Arrow shows doublets and arrow head shows singlet of Ndc80-mCherry. Scale bar = 5µm.

Journal of Cell Science • Supplementary information

Fig. S3

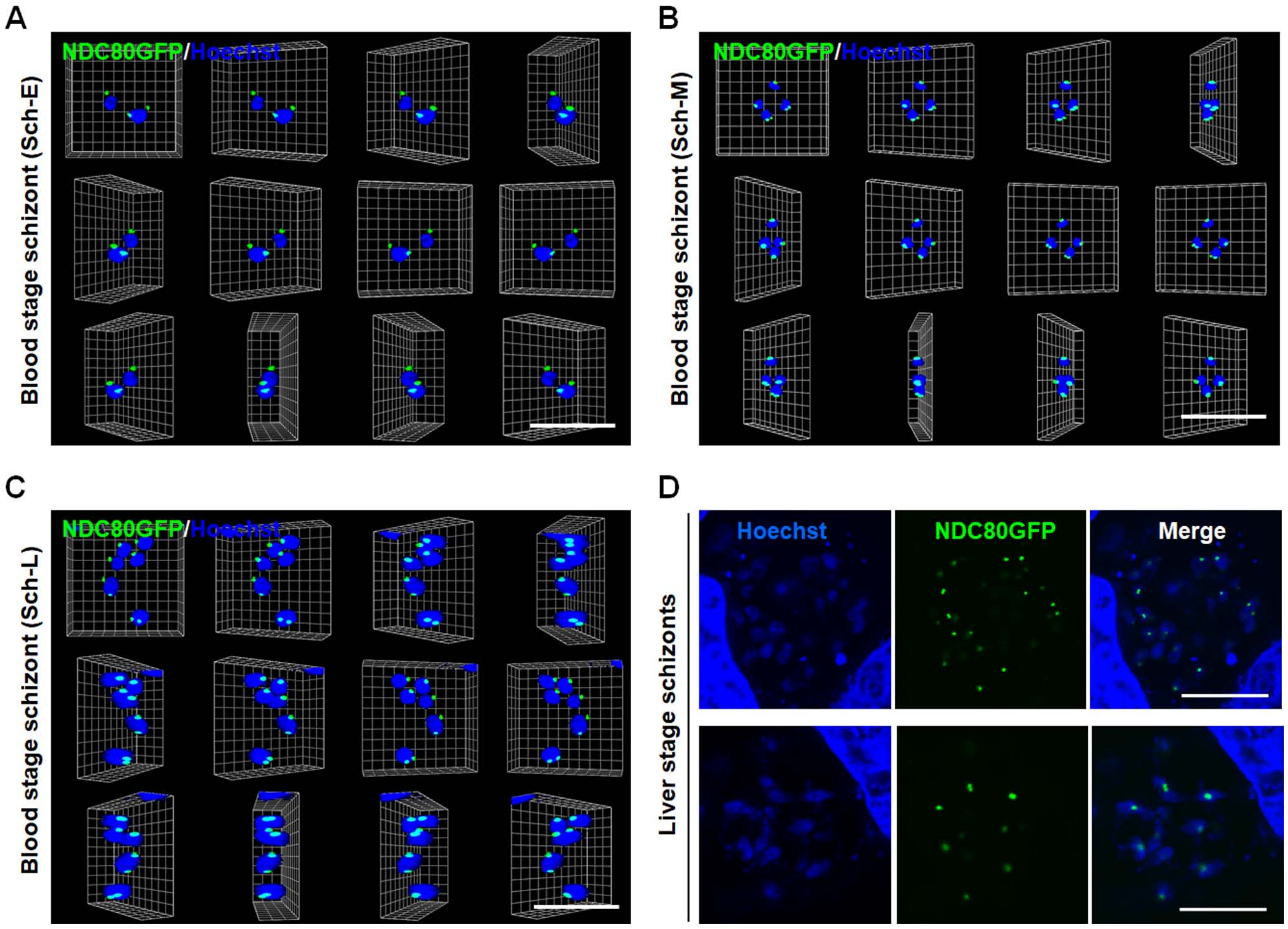

Fig. S3: Localization of *Plasmodium* NDC80-GFP during schizogony in blood and liver stages. Different views of three-dimensional super-resolution NDC80-GFP images during **(A)** early stage schizont, **(B)** middle stage schizont, **(C)** late stage schizont during blood stage schizogony ; Scale of the grid is 0.5 µm, **(D)** Expression of the protein was detected in liver schizonts by live cell imaging. Merge = DAPI and GFP. Scale bar = 5 µm.

Fig. S4

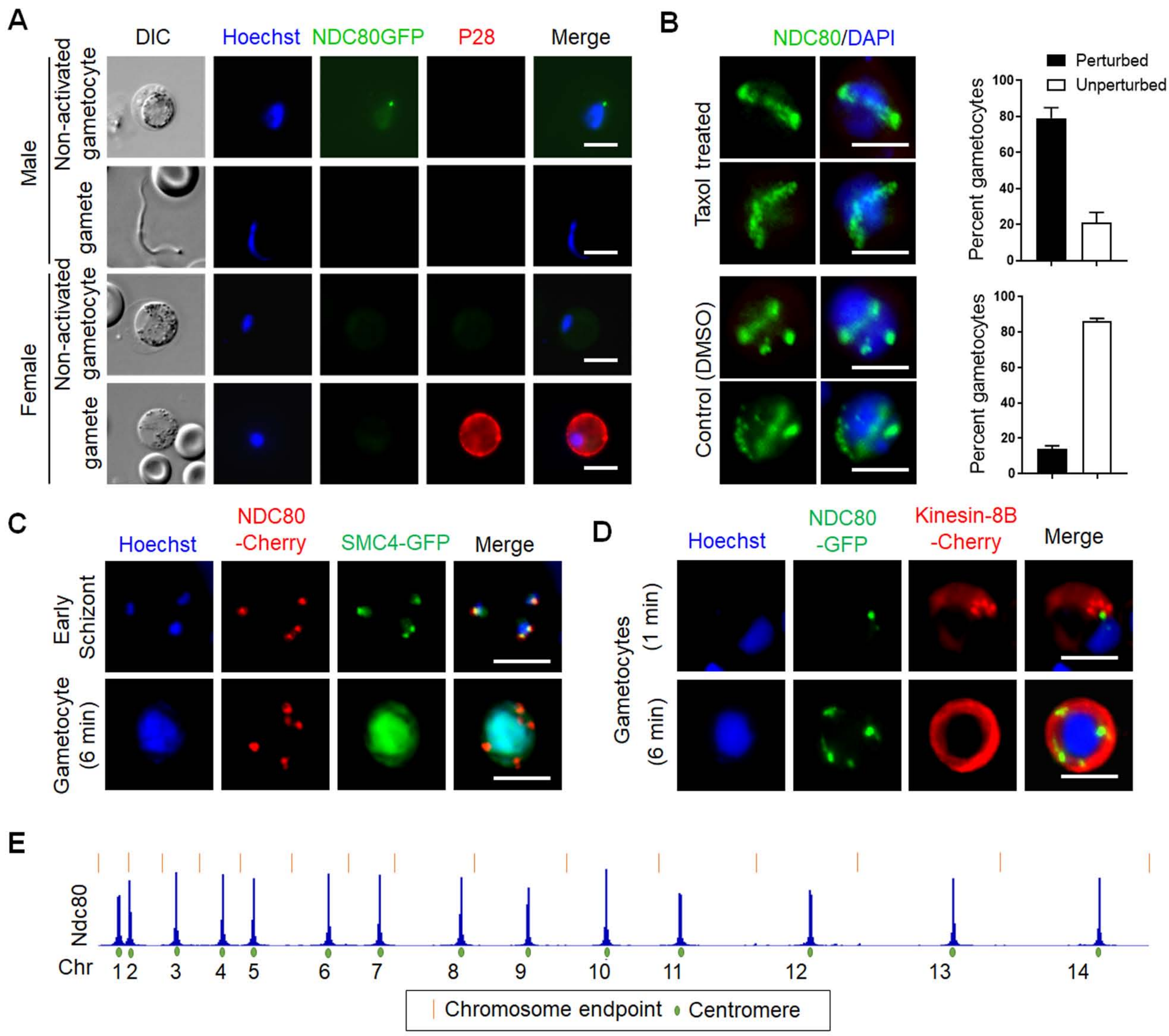

Fig. S4: NDC80 is not expressed in female gametocytes and gametes but shows centromeric location in male gametocytes. (A) Non-activated and activated gametocytes and gametes were examined by live cell imaging (100x magnification). DIC: differential interference contrast; Hoechst (blue, DNA); NDC80-GFP (green, GFP); P28 (red, cell surface marker of activated female gamete); Merge: Hoechst (blue, DNA), GFP (green) and P28 (red). **(B)** Male gametocytes treated with taxol (tubulin depolymerisation inhibitor) at 1 min post activation showing location of NDC80. DMSO was used as control. **(C)** The location of NDC80 (red) in relation to SMC4 (green), a kinetochore marker, in schizont and male gametocyte. **(D)** The location of NDC80 (green) in relation to kinesin-8B (red), an axonemal marker, in male gametocytes at 1 min- and 6 min- post activation. Scale bar = 5 µm. **(E)** Centromeric localization confirm by ChIP-seq analysis of NDC80-GFP profiles for all 14 chromosomes in gametocyte stage. Signals are plotted on a normalized read per million (RPM) basis.

Journal of Cell Science • Supplementary information

Fig. S5

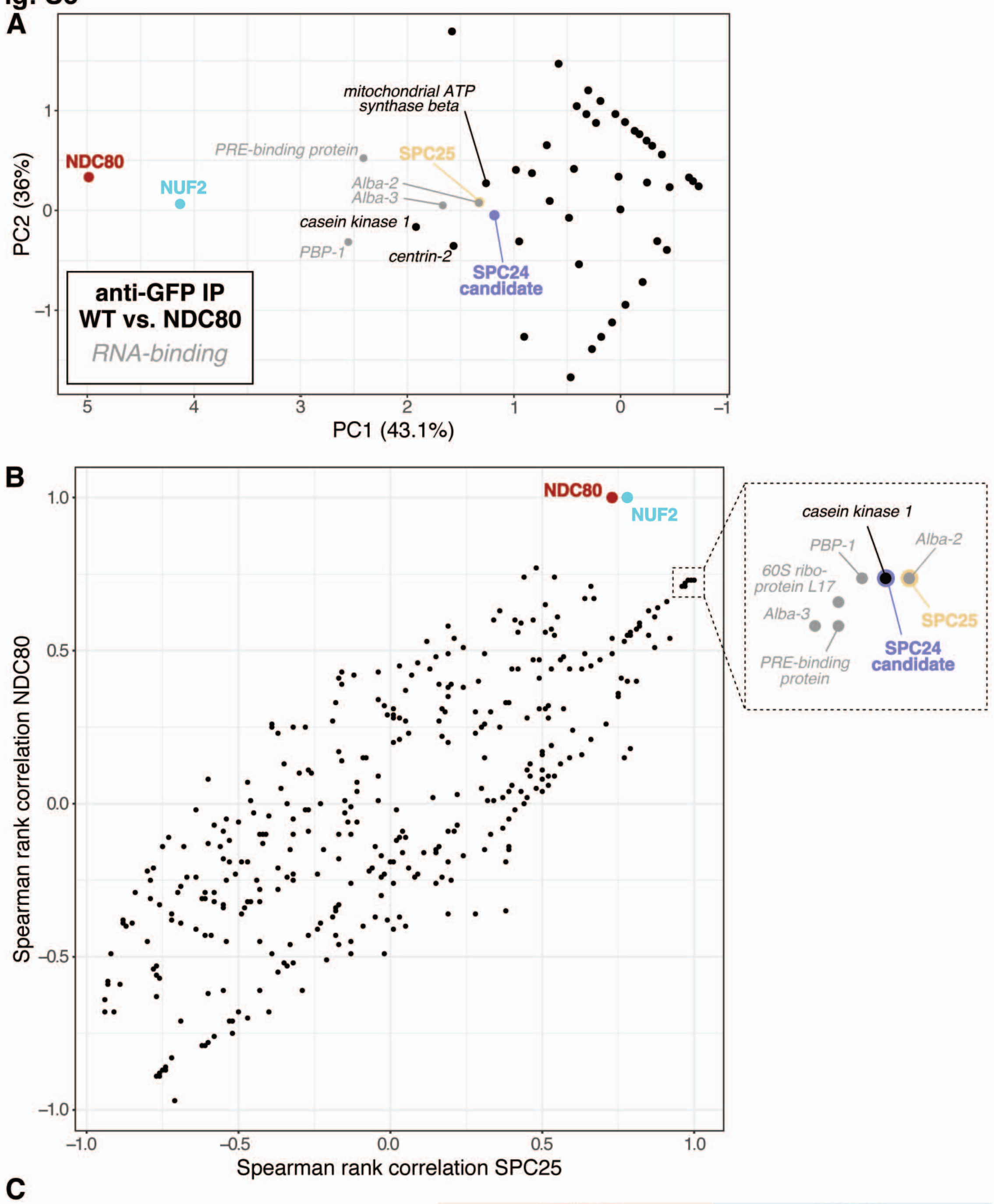

C

Identified Proteins	Accession Number	MW	Gametocytes 01_WTGFP	Gametocytes 02_NDC80_1	Gametocytes 03_NDC80_2	Schizonts 04_WTGFP	Schizonts 05_NDC80_1	Schizonts 06_NDC80_2
kinetochore protein NDC80	PBANKA_1115700	69 kDa	0	22	21	0	29	22
kinetochore protein NUF2	PBANKA_0414300	54 kDa	0	6	16	0	18	14
polyadenylate-binding protein 1	PBANKA_1439200	93 kDa	0	0	9	0	7	6
PRE-binding protein	PBANKA_1210200	111 kDa	0	0	4	0	9	8
DNA/RNA-binding protein Alba 3	PBANKA_1204400	12 kDa	0	0	4	0	5	4
DNA/RNA-binding protein Alba 2	PBANKA_1359200	24 kDa	0	0	4	0	2	5
kinetochore protein SPC25	PBANKA_1358800	26 kDa	0	0	4	0	2	5
conserved Plasmodium protein	PBANKA_1442300	96 kDa	0	0	4	0	2	4
casein kinase 1	PBANKA_0912100	38 kDa	0	0	6	0	4	5
centrin-2	PBANKA_1310400	19 kDa	0	0	8	0	0	7
ATP synthase beta, mitochondrial	PBANKA_1450300	58 kDa	0	0	4	0	0	8

Journal of Cell Science • Supplementary information

Fig. S5. Covariance, and correlation analysis of proteins detected in GFP pulldown experiments (WT vs. NDC80). (A) principal component analysis of natural log-transformed peptide counts of identified proteins in all NDC80-GFP pulldown experiments (schizonts and gametocytes) reported in this study (**Table S2**), excluding ribosomal proteins (often found as background hits). Subunits of the NDC80 complex are specifically highlighted, and colours correspond to those in Figure 6. Percentages indicate the variance explained by each component (PC1: 36%, PC2: 43.1, adding up to a total of 79.1%). Candidate proteins that show similar behaviour in different pulldown experiments are specifically annotated. Grey represents candidates that function in transcription and/or are known to interact with (m)RNA. **(B)** Plot of Spearman rank correlation of NDC80 (y-axis) and SPC25 (x-axis) with all proteins identified across control and NDC80-GFP pulldown experiments. Candidates were selected based on *R*>0.7 for both SPC25 and NDC80. NDC80 complex subunits are specifically highlighted, and colours are similar to panel B. **(C)** Table showing peptide count values in both control and NDC80-GFP pulldown experiments for candidates selected from the analyses in panel A and B.

Journal of Cell Science • Supplementary information

Fig. S6

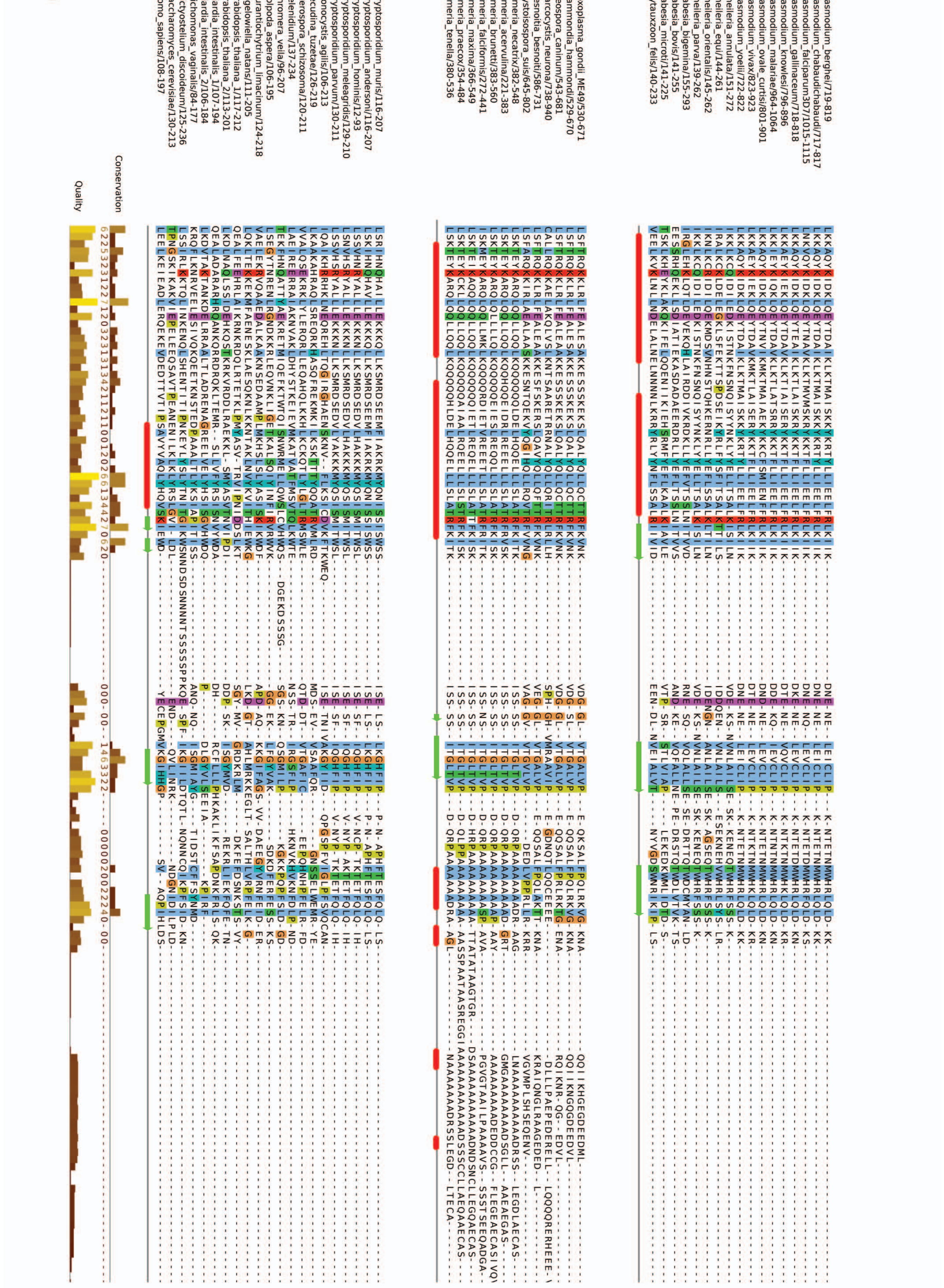

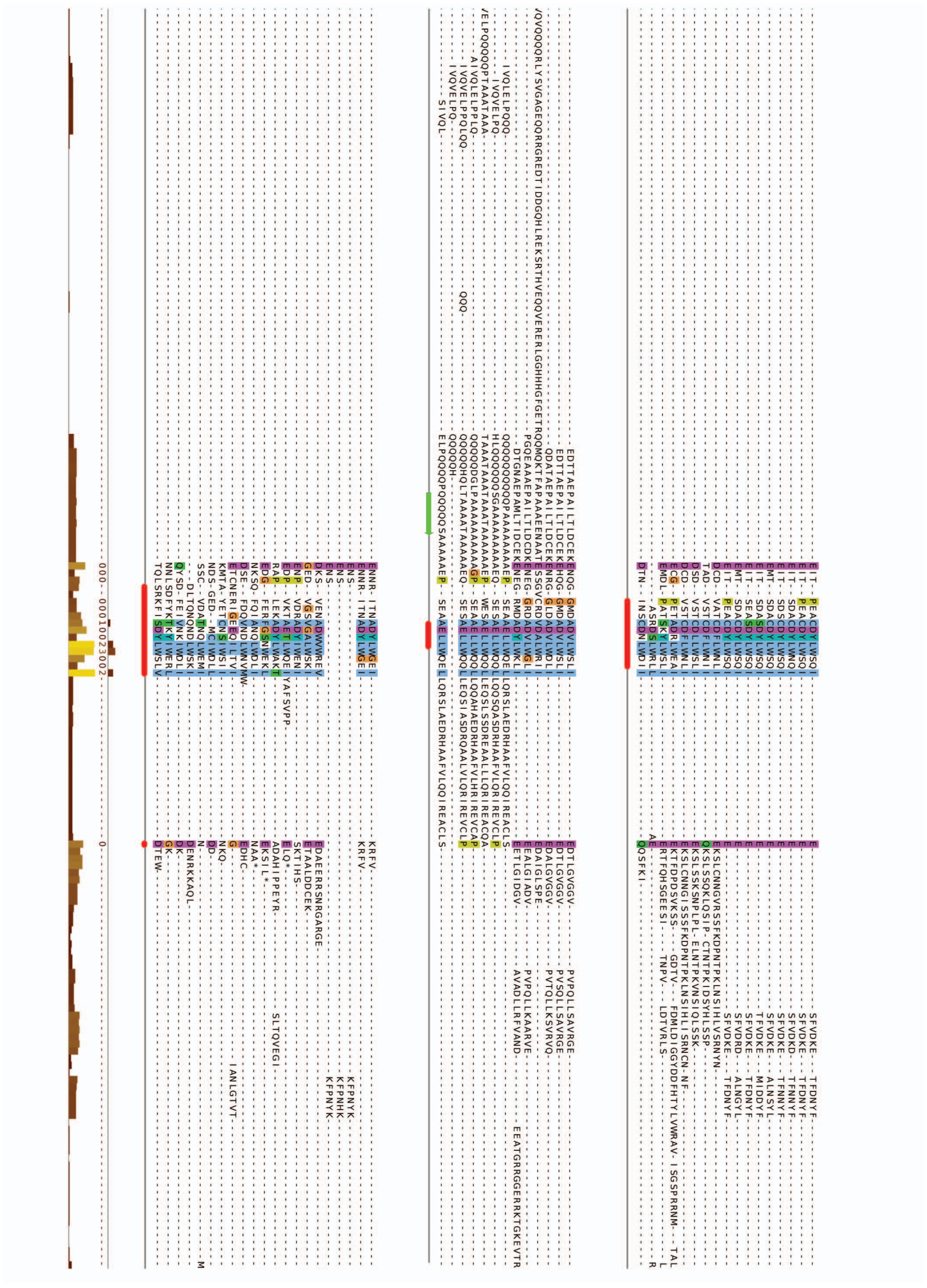

Fig. S6 Full-length alignment of RWD domains of different SPC24 candidate orthologs shown in Fig. 6C. RWD domain alignment of SPC24 candidate orthologs from our sequence database. Secondary structure of the three groups of orthologs (see **Fig. 6C**) is based on the HHsearch algorithm. Colours and conservation metric are based on the Clustal scheme as implemented in Jalview (Waterhouse et al., 2009).

Journal of Cell Science • Supplementary information

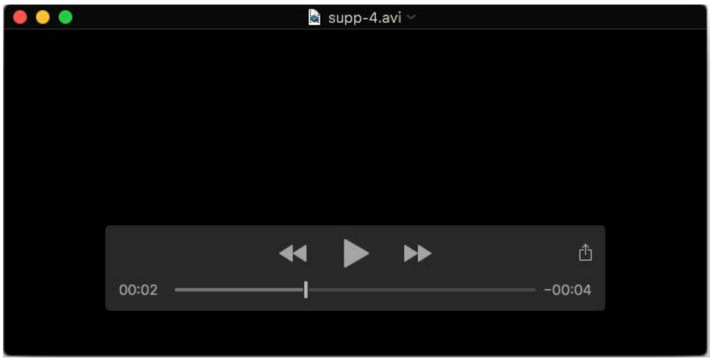

Movie 1. Three-dimensional visualization (3D rendered SIM Structured Illumination) of NDC80-GFP with respect to DAPI-stained nuclear DNA during early (SV1), middle (SV2) and late (SV3) stages of schizogony showing asynchronous division. Scale of the grid is 0.5 µm. See also Fig S3A, B and C.

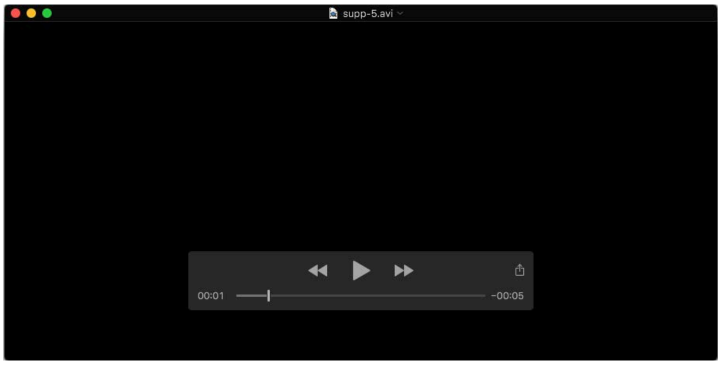

Movie 2. Three-dimensional visualization (3D rendered SIM Structured Illumination) of NDC80-GFP with respect to DAPI-stained nuclear DNA during early (SV1), middle (SV2) and late (SV3) stages of schizogony showing asynchronous division. Scale of the grid is 0.5 µm. See also Fig S3A, B and C.

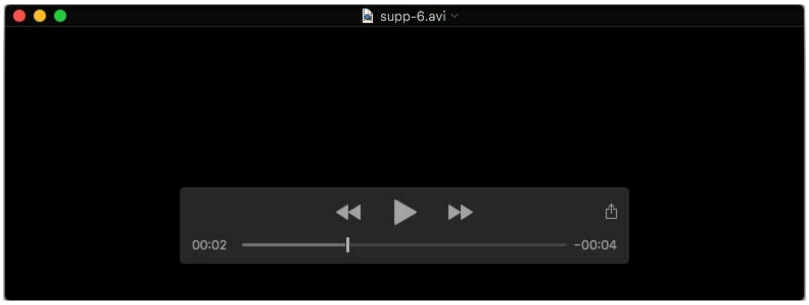

Movie 3. Three-dimensional visualization (3D rendered SIM Structured Illumination) of NDC80-GFP with respect to DAPI-stained nuclear DNA during early (SV1), middle (SV2) and late (SV3) stages of schizogony showing asynchronous division. Scale of the grid is 0.5 µm. See also Fig S3A, B and C.

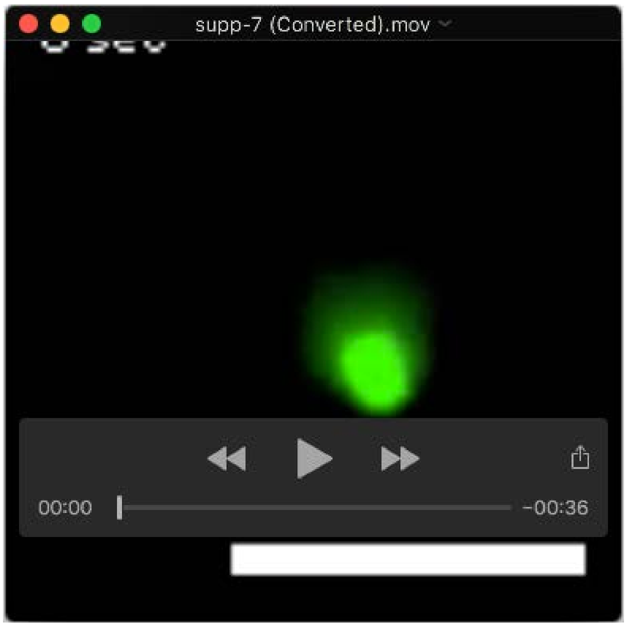

Movie 4. Time lapse video showing dynamics of NDC80-GFP during gametogenesis over 0 to 90 seconds after activation. Scale bar = 5 µm. See also Fig. 2B.

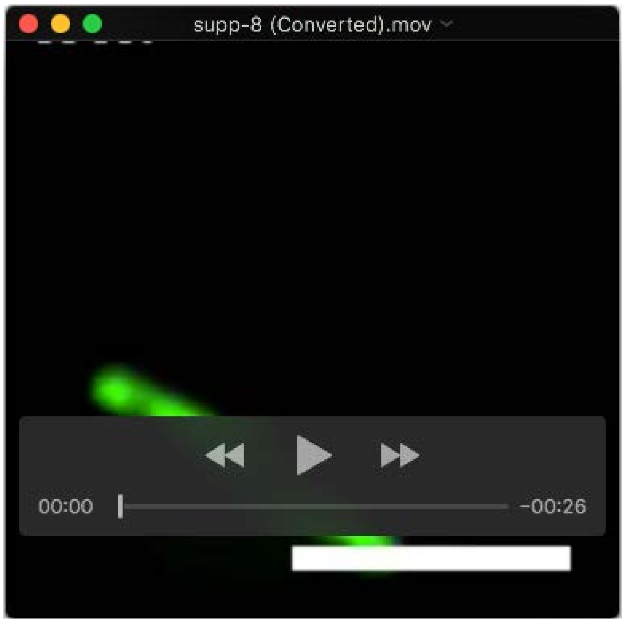

Movie 5. Time lapse video showing dynamics of NDC80-GFP during gametogenesis over 90 to 180 seconds after activation. Scale bar = 5 µm. See also Fig. 2C.

Journal of Cell Science • Supplementary information

Table S1: Primers used in this study.

Name	Sequence (5' to 3')	Notes
**T2591**	CCCCGGTACCGCGAATTCTAATAATAGGTTTAATC	KpnI site underlined
**T2592**	CCCCGGGCCCTTCAGTTACACTTTGATGTAAATTTTTATATAATTC	ApaI site underlined
**IntT259**	GATCTGGAAGAATCAATCAGAAAGAC	
**ol492**	ACGCTGAACTTGTGGCCG	For GFP line
**mCherry**	TTCAGCTTGGCGGTCTGGGT	For mCherry line

Table S2: List of main protein hits identified by mass spectrometry in the NDC80-GFP immunoprecipitation experiments. The two last columns represent Spearman rank correlation values of a particular gene product with NDC80 and SPC25 (see also **Fig. S5**). Colour coding corresponds with **Fig. 6** and **Fig. S5**. (separate excel sheet)

Click here to Download Table S2

Table S3: Sources of genomes and transcripts of the sequence database used for sequence similarity searches including hyperlinks and/or reference to papers. (separate excel sheet).

Click here to Download Table S3

Table S4: Presence/absence table of the NDC80 complex (NDC80, NUF2, SPC25, SPC24). Absences are denoted by '-' and presences by the length of the ortholog. (separate excel sheet)

Click here to Download Table S4

Journal of Cell Science • Supplementary information
