# Supplementary material for: Real-time dynamics of Plasmodium NDC80 reveals unusual modes of chromosome segregation during parasite proliferation
Source: J Cell Sci. 2020 Jun 30;134(5):jcs245753. doi: 10.1242/jcs.245753 (PMC7340582; doi:10.1242/jcs.245753)
Supplement: Reviewer comments [file joces-134-245753_review_history.pdf]

Journal of Cell Science

Real-time dynamics of *Plasmodium* NDC80 reveals unusual modes of chromosome segregation during parasite proliferation

Mohammad Zeeshan, Rajan Pandey, David J. P. Ferguson, Eelco C. Tromer, Robert Markus, Steven Abel, Declan Brady, Emilie Daniel, Rebecca Limenitakis, Andrew R. Bottrill, Karine G. Le Roch, Anthony A. Holder, Ross F. Waller, David S. Guttery and Rita Tewari
DOI: 10.1242/jcs.245753

Editor: David Glover

Review timeline
Original submission: 25 September 2019
Editorial decision: 4 November 2019
Resubmission: 26 February 2020
Editorial decision: 6 April 2020
First revision received: 23 April 2020
Accepted: 29 April 2020

Original submission

<u>Decision letter</u>

MS ID#: JOCES/2019/239574
MS TITLE: Real-time dynamics of Plasmodium NDC80 as a marker for the kinetochore during atypical mitosis and meiosis
AUTHORS: Rajan Pandey, Mohammad Zeeshan, David J.P. Ferguson, Robert Markus, Declan Brady, Emilie Daniel, Rebecca Stanway, Anthony A Holder, David S Guttery, and Rita Tewari
ARTICLE TYPE: Tools and Resources

Dear Dr. Tewari,

We have now reached a decision on the above manuscript.

To see the reviewers' reports and a copy of this decision letter, please go to: https://submit-jcs.biologists.org and click on the 'Manuscripts with Decisions' queue in the Author Area. (Corresponding author only has access)

As you will see from their reports, both reviewers raise a number of substantial criticisms that prevent me from accepting your paper for publication. Critically it is felt that there is insufficient advance in your paper for publication in JCS.

I am very sorry to give you such disappointing news, but we are currently under great pressure for space and it takes a very enthusiastic recommendation by the referees for a manuscript to be accepted.

I do hope you find the comments of the reviewers helpful in allowing you to revise the manuscript for submission elsewhere, and many thanks for sending your work to Journal of Cell Science.

© 2020. Published by The Company of Biologists under the terms of the Creative Commons Attribution License (https://creativecommons.org/licenses/by/4.0/).

Reviewer 1

Advance summary and potential significance to field

Kinetochores are the macromolecular protein complex that drives chromosome segregation in eukaryotes. Although extensive amounts of kinetochore research have been performed in select model organisms in opisthokonts (e.g. yeasts, worms, flies, humans), relatively little is known about kinetochores in divergent eukaryotes. Plasmodium, the causative agent of malaria, belongs to the SAR supergroup and is an interesting organism because previous bioinformatics studies failed to identify a number of kinetochore proteins, including Spc24, a subunit of the Ndc80 complex. It remains unclear whether Plasmodium actually lacks Spc24 or has a divergent molecule. In this manuscript, Pandey and colleagues tagged Ndc80 with fluorescent tags and examined its localization in different life stages of Plasmodium berghei using live cell imaging and superresolution microscopy.
Although this is the first study that analyzed the location of Ndc80, this is not the first study to look at kinetochore proteins in Plasmodium. Furthermore, although their imaging revealed the clustering of kinetochores, this phenomenon has been noted in previous studies (e.g. Hoeijmakers et al. 2012). Overall, although I agree that the created cell lines will likely to be a useful tool to analyze the dynamics of chromosome behavior or biochemical characterization of the Ndc80 complex in Plasmodium berghei, I do not feel this manuscript represents a significant advance in the field. I therefore cannot recommend its publication in the Journal of Cell Science.

Comments for the author

Other comment
- I felt it strange that the authors did not mention the papers by Verma and Surolia (Molecular & Biochemical Parasitology 2013 and Malaria Journal 2014) that studied CENH3 and CENP-Cin Plasmodium falciparum.

Reviewer 2

Advance summary and potential significance to field

Summary of the advance made in this paper and its potential significance to the field:
Plasmodium falciparum, the parasite causing malaria, presents a complex life cycle that includes two unique sets mitoses and one single stage of meiosis, all carried out in two separate organisms (i.e. the vector and the host). Despite the advances in our understanding of how chromosome segregation is faithfully performed in human cells and in other model organisms, the molecular details remain unclear. In this new study, the authors developed a new tool to allow fluorescence-based localisation studies of the Ndc80 complex of P. falciparum (pfNdc80). Live-cell and 3D-SIM imaging, immunofluorescence studies and ultrastructural analysis show that pfNdc80 is located in a single cluster of subdiffraction limit, but present complex dynamics during the life cycle of the parasite. Despite the lack of more mechanistic insights in the function and organisation of the Ndc80 complex in P. falciparum, this study characterises a new tool that has the potential to be used for more mechanism-driven studies to study the life cycle of plasmodium. Considering the lack of data on the pathogen plasmodium, and the difficulty in studying the pathogen, I would consider this advance reported as significant one. However, the manuscript should consider a few key points (listed below) prior to publication.

Comments for the author

Major comments that should be addressed first:
1) Fig 2D-E are used to claim that fpNdc80-GFP signal is not overlapping with alpha-tubulin (2D) and centrin signals (2E). This is convincing for 2E but not for 2D. Since Ndc80 has been extensively studied and it is clear that it presents an evolutionarily conserved role in microtubule binding during cell division, this claim should be carefully revised. First, in Fig 2D (lower subpanel), a partial overlap of Ndc80-GFP and alpha-tubulin is clearly visible. One possibility would be to co-localise Ndc80-GFP and tubulin using super-resolution, to resolve subdiffraction distances.

© 2020. Published by The Company of Biologists under the terms of the Creative Commons Attribution License (https://creativecommons.org/licenses/by/4.0/).

Alternately they could count the number of instances such a partial overlap was seen or not seen. Second, Fig 3D clearly shows that kinetochores are clearly connected to the microtubule emanated to the nuclear poles (K and NP labels in the image). If Ndc80 is a kinetochore component also in P. falciparum (as it is suggested by other evolutionary studies, like van Hoof et al. 2017), then its localisation on the spindle during mitosis should be further characterised.

2) This paper presents good quality light-microscopy images but some co-localisation studies would help place pfNdc80 more clearly at the KTs. Since Mis12, Nuf2 and CENP-A are conserved in plasmodium, it would be useful to have at least one them tagged as and co-localised with Ndc80 to map whether the architecture of the outer kinetochore is still conserved in P. falciparum. If this is not possible, then they could colocalise with a live-cell DNA marker for the movies (SiRDNA to see if the colocalisation remains static and not dynamic as for a microtubule binding protein). Alternately, the authors could perform nocodazole treatment (to depolymerise microtubules) and see if the punctae is still clearly visible in the absence of microtubule signal.

Minor comments:
1) In introduction, the first section of citations is not linked correctly in the text; all the paper cited refer to reviews on the kinetochore structure and not to more general overviews of mitosis.

2) In Fig5A, the Hoechst stain in the 14 dpi subpanel is not convincing. The signal appears to localise with the membrane of the oocyst, whereas the DIC clearly suggests the more central and tri-lobed signal should be the nuclei of the sporozoites. The subpanel should be substituted or commented in the text if representative of all the 14 dp1 oocysts.

3) In Fig5B, the scale bar of the ultra-structural analysis is not indicated in the legend. Movies need scale bar as well.

4) In the schizonts, Fig S3 doublets of Ndc80-GFP are visible - this could be highlighted with arrows or as a cropped magnified image.

5) SV1, 2 and 3 were unclear for me.

6) Videos will benefit from timestamps.

Resubmission

<u>Author response to reviewers' comments</u>

Manuscript JOCES/2019/239574, reviewers' comments and our response.

Reviewer 1 Advance Summary and Potential Significance to Field:
Kinetochores are the macromolecular protein complex that drives chromosome segregation in eukaryotes. Although extensive amounts of kinetochore research have been performed in select model organisms in opisthokonts (e.g. yeasts, worms, flies, humans), relatively little is known about kinetochores in divergent eukaryotes. Plasmodium, the causative agent of malaria, belongs to the SAR supergroup and is an interesting organism because previous bioinformatics studies failed to identify a number of kinetochore proteins, including Spc24, a subunit of the Ndc80 complex. It remains unclear whether Plasmodium actually lacks Spc24 or has a divergent molecule. In this manuscript, Pandey and colleagues tagged Ndc80 with fluorescent tags and examined its localization in different life stages of Plasmodium berghei using live cell imaging and superresolution microscopy. Although this is the first study that analyzed the location of Ndc80, this is not the first study to look at kinetochore proteins in Plasmodium. Furthermore, although their imaging revealed the clustering of kinetochores, this phenomenon has been noted in previous

© 2020. Published by The Company of Biologists under the terms of the Creative Commons Attribution License (https://creativecommons.org/licenses/by/4.0/).

studies (e.g. Hoeijmakers et al. 2012). Overall, although I agree that the created cell lines will likely to be a useful tool to analyze the dynamics of chromosome behavior or biochemical characterization of the Ndc80 complex in Plasmodium berghei, I do not feel this manuscript represents a significant advance in the field. I therefore cannot recommend its publication in the Journal of Cell Science.
We show here that the Plasmodium genome does encode Spc24, but the gene is not annotated correctly in databases. We identified the protein (PBANKA_1442300) bound to Ndc80-GFP by mass spectroscopy. Bioinformatic analysis confirmed that PBANKA_1442300 has significant homology with Spc24 (Fig. 6).

Although there was a previous study of Plasmodium kinetochore proteins expressed during blood stage schizogony, this is the first study of parasite stages in the mosquito. It is also the first study using live cell imaging, fixed cell immunofluorescence and electron microscopy to examine the whole parasite life cycle.

We think that the revised manuscript represents a significant advance in the field. It examines aspects of mitosis within single cells that display multiple asynchronous nuclear divisions (schizogony), or rapid replication and chromosome segregation within a single nucleus (during gametogenesis), at different stages of the Plasmodium life cycle. It defines the kinetochore protein complex by both proteomic and bioinformatic studies, and it examines the co-location of this complex with other proteins associated with chromosomes and microtubules in either the nucleus or the cytoplasm.

Reviewer 1 Comments for the Author:
Other comment
I felt it strange that the authors did not mention the papers by Verma and Surolia Molecular & Biochemical Parasitology 2013 and Malaria Journal 2014) that studied CENH3 and CENP-C in Plasmodium falciparum.
This work is now cited in the discussion section.

Reviewer 2 Advance Summary and Potential Significance to Field:
Summary of the advance made in this paper and its potential significance to the field: Plasmodium falciparum, the parasite causing malaria, presents a complex life cycle that includes two unique sets mitoses and one single stage of meiosis, all carried out in two separate organisms (i.e. the vector and the host). Despite the advances in our understanding of how chromosome segregation is faithfully performed in human cells and in other model organisms, the molecular details remain unclear. In this new study, the authors developed a new tool to allow fluorescence-based localisation studies of the Ndc80 complex of P. falciparum (pfNdc80). Live-cell and 3D-SIM imaging, immunofluorescence studies and ultrastructural analysis show that pfNdc80 is located in a single cluster of subdiffraction limit, but present complex dynamics during the life cycle of the parasite. Despite the lack of more mechanistic insights in the function and organisation of the Ndc80 complex in P. falciparum, this study characterises a new tool that has the potential to be used for more mechanism-driven studies to study the life cycle of plasmodium. Considering the lack of data on the pathogen plasmodium, and the difficulty in studying the pathogen, I would consider this advance reported as significant one. However, the manuscript should consider a few key points (listed below) prior to publication.

Reviewer 2 Comments for the Author:
Major comments that should be addressed first:
1) Fig 2D-E are used to claim that fpNdc80-GFP signal is not overlapping with alpha-tubulin (2D) and centrin signals (2E). This is convincing for 2E but not for 2D. Since Ndc80 has been extensively studied and it is clear that it presents an evolutionarily conserved role in microtubule binding during cell division, this claim should be carefully revised. First, in Fig 2D (lower subpanel), a partial overlap of Ndc80-GFP and alpha-tubulin is clearly visible. One possibility would be to co-localise Ndc80-GFP and tubulin using super-resolution, to resolve subdiffraction distances. Alternately they could count the number of instances such a partial overlap was seen or not seen. Second, Fig 3D clearly shows that kinetochores are clearly connected to the microtubule emanated to the nuclear poles (K and NP labels in the image). If Ndc80 is a kinetochore component also in P. falciparum (as it is suggested by other evolutionary studies, like van Hoof et al. 2017), then its localisation on the spindle during mitosis should be further characterised.

© 2020. Published by The Company of Biologists under the terms of the Creative Commons Attribution License (https://creativecommons.org/licenses/by/4.0/).

We have clarified the text. The Ndc80-GFP signal does partially overlap with the tubulin signal in early stages of schizogony, but not in later stages (Fig. 2D). The quantification of overlap requested by the reviewer is now provided in the figure. We have also provided additional data to locate Ndc80 on the spindle during early male gametogenesis, and additional colocalization studies of Ndc80 with the condensin marker SMC4 and the cytoplasmic axonemal marker Kinesin-8B (See Figure S4). The location of Ndc80 does not overlap with that of centrin in either early or late stages of schizogony (Figure2E).

2) This paper presents good quality light-microscopy images but some co-localisation studies would help place pfNdc80 more clearly at the KTs. Since Mis12, Nuf2 and CENP-A are conserved in plasmodium, it would be useful to have at least one them tagged as and co- localised with Ndc80 to map whether the architecture of the outer kinetochore is still conserved in P. falciparum. If this is not possible, then they could colocalise with a live-cell DNA marker for the movies (SiRDNA to see if the colocalisation remains static and not dynamic as for a microtubule binding protein). Alternately, the authors could perform nocodazole treatment (to depolymerise microtubules) and see if the punctae is still clearly visible in the absence of microtubule signal.

We provide data to show colocalization of Ndc80 and the condensin subunit SMC4, which both have a centromeric location in early schizonts (see Figure S4C). This location was confirmed by ChIPseq analysis (Figure S4E). The proteomic data also show that the architecture of the outer kinetochore is conserved because Ndc80-GFP is in complex with other components of the outer kinetochore (NUF2, SPC24 and SPC25) (Figure 6B). We also show the effect of taxol treatment on NDC80 location (Fig S4B); taxol stabilises microtubular filaments and inhibits separation of the kinetochores towards the nuclear poles.

Minor comments:
1) In introduction, the first section of citations is not linked correctly in the text; all the paper cited refer to reviews on the kinetochore structure and not to more general overviews of mitosis.
The introduction has been modified to include more general information about kinetochore structure.

2) In Fig5A, the Hoechst stain in the 14 dpi subpanel is not convincing. The signal appears to localise with the membrane of the oocyst, whereas the DIC clearly suggests the more central and tri-lobed signal should be the nuclei of the sporozoites. The subpanel should be substituted or commented in the text if representative of all the 14 dpi oocysts.
The Figure has been modified and the oocyst image at 14 dpi has been substituted.

3) In Fig5B, the scale bar of the ultra-structural analysis is not indicated in the legend. Movies need scale bar as well.
Scale bars are provided now for all images and movies, and indicated in the legends.

4) In the schizonts, Fig S3 doublets of Ndc80-GFP are visible - this could be highlighted with arrows or as a cropped magnified image.
We have cropped and magnified the images, and added an arrow and arrowhead for clarification.

5) SV1, 2 and 3 were unclear for me.
These movies are now explained more fully in the figure legends

6) Videos will benefit from timestamps.
"Thank you for your suggestion, however videos SV1-3 are single time points, showing a 3D representation of the images. The grid in the background is plotted in order to help 3D visualization as well as it serves as a scale. The size of the squares is 0.5 um."

© 2020. Published by The Company of Biologists under the terms of the Creative Commons Attribution License (https://creativecommons.org/licenses/by/4.0/).

First decision letter

MS ID#: JOCES/2020/245753

MS TITLE: Real-time dynamics of Plasmodium NDC80reveals unusual modes of chromosome segregation during parasite proliferation

AUTHORS: Rita Tewari, Mohammad Zeeshan, Rajan Pandey, David JP Ferguson, Eelco C Tromer, Robert Markus, Steven Abel, Declan Brady, Emilie Daniel, Rebecca Limenitakis, Andrew R Bottrill, Karine G LeRoch, Anthony A Holder, Ross F Waller, and David S Guttery
ARTICLE TYPE: Research Article

We have now reached a decision on the above manuscript.

To see the reviewers' reports and a copy of this decision letter, please go to: https://submit-jcs.biologists.org and click on the 'Manuscripts with Decisions' queue in the Author Area. (Corresponding author only has access to reviews.)

As you will see, the reviewers gave favourable reports but raised some critical points that will require amendments to your manuscript. I hope that you will be able to carry these out, because I would like to be able to accept your paper.

We are aware that you may currently be unable to access the lab to undertake experimental revisions. If it would be helpful, we encourage you to contact us to discuss your revision in greater detail. Please send us a point-by-point response indicating where you are able to address concerns raised (either experimentally or by changes to the text) and where you will not be able to do so within the normal timeframe of a revision. We will then provide further guidance. Please also note that we are happy to extend revision timeframes as necessary.

Please ensure that you clearly highlight all changes made in the revised manuscript. Please avoid using 'Tracked changes' in Word files as these are lost in PDF conversion.

I should be grateful if you would also provide a point-by-point response detailing how you have dealt with the points raised by the reviewers in the 'Response to Reviewers' box. Please attend to all of the reviewers' comments. If you do not agree with any of their criticisms or suggestions please explain clearly why this is so.

Reviewer 1

Advance summary and potential significance to field

My main problem on the initial manuscript was that, although the authors created a nice tool, I did not feel the manuscript provided significant progress. Previous bioinformatics studies failed to identify Spc24 in Plasmodium but it remained unclear whether Plasmodium actually lacks Spc24 or has a divergent molecule. In the revised manuscript, the authors performed pulldown of Ndc80-GFP and mass spectrometry as well as advanced bioinformatics, which led to the identification of a divergent Spc24 homolog. I view it as an important progress because it shows the limitation of bioinformatic approach to identify divergent homologs and shows the importance of taking web-lab approach (and subsequent advanced bioinformatics such as HMM-HMM comparison) to identify a true catalog of kinetochore proteins in a given organism. I now support publication of this manuscript in the Journal of Cell Science.

Comments for the author

Minor comments: I found a number of typos throughout the manuscript. I suggest that the authors perform a carful proofreading before publication. Below are some examples.
- Page 4: rGametogenesis

© 2020. Published by The Company of Biologists under the terms of the Creative Commons Attribution License (https://creativecommons.org/licenses/by/4.0/).

- Page 5: with very a divergent
- page 12 and 15: GPF

Reviewer 2

Advance summary and potential significance to field

Using a kinetochore marker Ndc80, the authors indicate different modes of chromosome segregation during proliferative stages of the malaria parasite life-cycle.

Comments for the author

No major revisions needed.

I would suggest toning down the claim for discovering a divergent Spc24 as there is no direct evidence for this (for example, tagging Spc24 with mCherry/DsRed to colocalise with Ndc80-GFP or copurification with Spc25 are missing). It's also unclear why Spc24 is so divergent but not Spc25. These could be addressed in the discussion: Is plasmodium Spc25 four or five times longer compared to eukaryotic Spc25? or is this unique to plasmodium Spc24 alone?

Minor points:
1. The movie did not open for me - so I am unable to comment about the movie. If not timestamps, please include stamps of z-positions to help readers.
2. I could not find "Supplementary Sequence File 1".
3. I could not find the database referred to below under Supplementary Table-2
"We, therefore, constructed a large sequence database consisting of 64 apicomplexan and other eukaryotic genomes and transcriptomes
(Supplementary Table 2), "
5. Several typos continue to exist: for example, page 9 "NDC-GFP fluorescence ", page 12
"Using a combination of GPF-pulldown",

First revision

Author response to reviewers' comments

Response to Reviewers' comments and suggestions

We are thankful to both reviewers for their time and effort, and their endorsement of our study. We address below their comments to further improve the manuscript.

Reviewer 1 Comments for the author
We are pleased with the reviewer's comment: "I now support publication of this manuscript in the Journal of Cell Science".

Minor comments:
I found a number of typos throughout the manuscript. I suggest that the authors perform a carful proofreading before publication.
Below are some examples.
-Page 4: rGametogenesis
-Page 5: with very a divergent
-page 12 and 15: GPF
We apologise for the presence of the typos in the manuscript. We have now checked the text carefully and corrected the errors.

Reviewer 2 Comments for the author

© 2020. Published by The Company of Biologists under the terms of the Creative Commons Attribution License (https://creativecommons.org/licenses/by/4.0/).

No major revisions needed.
I would suggest toning down the claim for discovering a divergent Spc24 as there is no direct evidence for this (for example, tagging Spc24 with mCherry/DsRed to colocalise with Ndc80- GFP or copurification with Spc25 are missing).

We agree that additional tagging experiments would help confirm our identification of a divergent SPC24, in particular the reciprocal pulldown for SPC24 and co-localisation with NDC80 to provide proof for functional identity. At the moment we cannot provide direct evidence for kinetochore localisation of the SPC24 candidate due to current Covid-19 restrictions that do not permit laboratory work. We believe that there is a strong case to interpret these sequences as bona fide SPC24 orthologs, but we have toned down our claims for the discovery of SPC24 orthologs by qualifying the description (for example, “candidate ortholog”). We have also extended the text in the discussion to clarify our identification of candidate SPC24 sequences orthologs. Future efforts will focus on verifying the kinetochore localisation of the SPC24 candidate and its presence in the NDC80 complex.

It's also unclear why Spc24 is so divergent but not Spc25. These could be addressed in the discussion: Is plasmodium Spc25 four or five times longer compared to eukaryotic Spc25? or is this unique to plasmodium Spc24 alone?

We have clarified the section in the discussion, where we speculate on the significance of the sequence extensions of the candidate SPC24 orthologs in Plasmodium spp. and Coccidia. We have now extended this part of the discussion to cover the other subunits of the NDC80 complex in Apicomplexa. Previous studies (van Hooff et al. 2017) had shown that SPC24 was among the most divergent kinetochore genes. Since the coiled-coils cannot be used as a reliable way to detect orthologs, with SPC24, one is left with a very small RWD domain to search for homologs. In our view the limited number of positions in the domain is the primary reason why detection of SPC24 is such an elaborate undertaking at present.
The extended length of SPC24 candidate orthologs (3-4 times), specifically in Plasmodium spp. and Coccidia is odd, but it seems to be mostly due to an N-terminal extension of the coiled-coil region, and some insertions into loops of the RWD domain in Coccidia. While SPC25 in Coccidia is twice as long (similar pattern of coiled-coil extension), possible mirroring the extension of the SPC24 candidate orthologs in these lineages, SPC25 orthologs in other Apicomplexa show a normal eukaryotic length distribution (~200-250aa). The SPC24 extended length in Plasmodiidae is therefore striking as SPC25 is a normal length. We speculate that the long SPC24 in Plasmodium might provide an additional binding site on the NDC80 complex for novel interactors, possible specific to Plasmodium spp. An alternative option is that the N-terminal coiled coils of SPC24 have a larger binding interface with the coiled-coils of NUF2 and NDC80, possibly providing additional rigidity to the NDC80 super structure.

Minor points: 1. The movie did not open for me - so I am unable to comment about the movie. If not timestamps, please include stamps of z-positions to help readers.
We apologise that the movie could not be opened by the reviewer. We have checked it again and, as suggested by reviewer, we also provide the stamps of Z-position for videos SV1 to 3 in Fig S3A to C. Time stamps have already been provided for SV4 in Fig 3B and for SV5 in Fig 3C.

2. I could not find "Supplementary Sequence File 1".
We are very sorry for this omission, as we thought we had attached it on the editorial manager system. We have now provided one single text file containing all detected (candidate) orthologs from this study for all four subunits of the NDC80 complex.

3. I could not find the database referred to below under Supplementary Table-2 "We, therefore, constructed a large sequence database consisting of 64 apicomplexan and other eukaryotic genomes and transcriptomes (Supplementary Table 2), "
We apologise for the confusion as this database should have been referenced as supplementary Table S3. This table contains links and references to the sources for the amino acid sequences that are contained within this manually curated and collated database. Since the database is of considerable size, we are not able make this publicly available in a useful format, and the dataset is therefore not directly available for public use. However, based on the information provided it is feasible to recreate the sequence database that was used for this study. To further clarify that the

© 2020. Published by The Company of Biologists under the terms of the Creative Commons Attribution License (https://creativecommons.org/licenses/by/4.0/).

supplementary table contains only references and links to sources for the sequence database, we have now specifically mention this in the reference to supplementary Table S3.

5. Several typos continue to exist: for example, page 9 "NDC-GFP fluorescence ", page 12 "Using a combination of GPF-pulldown",
We apologise for these typos. We we have now carefully checked and corrected the manuscript.

Second decision letter

MS ID#: JOCES/2020/245753

MS TITLE: Real-time dynamics of Plasmodium NDC80 reveals unusual modes of chromosome segregation during parasite proliferation

AUTHORS: Mohammad Zeeshan, Rajan Pandey, David JP Ferguson, Eelco C Tromer, Robert Markus, Steven Abel, Declan Brady, Emilie Daniel, Rebecca Limenitakis, Andrew R Bottrill, Karine G LeRoch, Anthony A Holder, Ross F Waller, David S Guttery, and Rita Tewari
ARTICLE TYPE: Research Article

I am happy to tell you that your manuscript has been accepted for publication in Journal of Cell Science, pending standard ethics checks.

© 2020. Published by The Company of Biologists under the terms of the Creative Commons Attribution License (https://creativecommons.org/licenses/by/4.0/).
